# Identifying Antidepressant Effects of Brain-Derived Neurotrophic Factor and IDO1 in the Mouse Model Based on RNA-Seq Data

**DOI:** 10.3389/fgene.2022.890961

**Published:** 2022-05-30

**Authors:** Jing Ren, Chenyang Li, Songren Wei, Yanjun He, Peng Huang, Jiangping Xu

**Affiliations:** ^1^ Department of Neuropharmacology and Novel Drug Discovery, School of Pharmaceutical Sciences, Southern Medical University, Guangzhou, China; ^2^ Students Affairs Division, Zhujiang Hospital of Southern Medical University, Guangzhou, China; ^3^ The Second School of Clinical Medicine, Southern Medical University, Guangzhou, China; ^4^ Emergency Department, Affiliated Foshan Maternity & Child Healthcare Hospital, Southern Medical University, Foshan, China; ^5^ Women and Children Medical Research Center, Affiliated Foshan Maternity & Child Healthcare Hospital, Southern Medical University, Foshan, China

**Keywords:** depression, RNA-seq, pathway enrichment analysis, protein–protein interaction (PPI) network, brain-derived neurotrophic factor (BDNF), indoleamine 2,3-dioxygenase 1 (IDO1)

## Abstract

Deletion of brain-derived neurotrophic factor (BDNF) and upregulation of indoleamine 2,3-dioxygenase 1 (IDO1) are associated with depression severity in animals. The neurotransmitter hypothesis of depression at the transcriptomic level can be tested using BDNF- and IDO1-knockout mouse models and RNA-seq. In this study, BDNF^+/−^, IDO1^−/−^, and chronic ultra-mild stress (CUMS)-induced depression mouse models and controls were developed, and the differentially expressed genes were analyzed. Furthermore, the ceRNA package was used to search the lncRNA2Target database for potential lncRNAs. Finally, a protein–protein interaction (PPI) network was constructed using STRINGdb. By comparing the control and CUMS model groups, it was found that pathway enrichment analysis and ceRNA network analysis revealed that most differentially expressed genes (DEGs) were associated with protection of vulnerable neuronal circuits. In addition, we found the enriched pathways were associated with nervous system development and synapse organization when comparing the control and BDNF^+/−^model groups. When replicating the neurotransmitter disruption features of clinical patients, such comparisons revealed the considerable differences between CUMS and knockdown BDNF models, and the BDNF^+/−^model may be superior to the classic CUMS model. The data obtained in the present study implicated the potential DEGs and their enriched pathway in three mouse models related to depression and the regulation of the ceRNA network-mediated gene in the progression of depression. Together, our findings may be crucial for uncovering the mechanisms underlying the neurotransmitter hypothesis of depression in animals.

## Introduction

Depression is a common mental disorder characterized by high morbidity and suicidal risk (Auerbach et al., 2018; Devendorf et al., 2020). Previous studies have shown that depression is a complex disorder involving multiple genes (Fan et al., 2020; Kang et al., 2020). The brain-derived neurotrophic factor (BDNF) gene, which is widely involved in emotion and cognition, has neurotrophic effects and modulates neuron regeneration, synaptic plasticity, and dendritic growth (Kowianski et al., 2018; Lima Giacobbo et al., 2019). Several studies have shown that BDNF is involved in the pathogenesis of neuropsychiatric diseases (Lima Giacobbo et al., 2019; Colucci-D’amato et al., 2020). Chronic social defeat stress in a rat model of depression has revealed a significant reduction of BDNF levels in the hippocampus and prefrontal cortex (Amidfar et al., 2018).

Increasing studies have shown that rats that have been deprived of maternal care during their young stage exhibit reduced hippocampal BDNF levels, short- and long-term deficits in aversion, and recognition memory, as well as cognitive flexibility (Menezes et al., 2020). Environmental enrichment interventions restore the levels of hippocampal BDNF in rats and protect their memory and cognitive flexibility (Zhang et al., 2020). Furthermore, the reduced level of BDNF has been associated with anhedonia (Dong et al., 2018) which is the main symptom of depression. The deletion of brain-derived neurotrophic factor (BDNF) and upregulation of indoleamine 2,3-dioxygenase 1 (IDO1) are associated with depression severity in animals. The neurotransmitter hypothesis of depression at the transcriptomic level can be tested using BDNF- and IDO1-knockout mouse models and RNA-seq. In this study, BDNF^+/−^, IDO1^−/−^, and chronic ultra-mild stress (CUMS)-induced depression mouse models and controls were developed, and the differentially expressed genes were analyzed. Furthermore, the ceRNA package was used to search the lncRNA2Target database for potential lncRNAs. Finally, a protein–protein interaction (PPI) network was constructed using STRINGdb. By comparing the control and CUMS model groups, it was found that pathway enrichment analysis and ceRNA network analysis revealed that most differentially expressed genes (DEGs) were associated with the protection of vulnerable neuronal circuits. In addition, we found the enriched pathways were associated with nervous system development and synapse organization when comparing the control and BDNF^+/−^model groups. When replicating the neurotransmitter disruption features of clinical patients, such comparisons revealed the considerable differences between CUMS and knockdown BDNF models, and the BDNF^+/−^model may be superior to the classic CUMS model. The data obtained in the current study implicated the potential DEGs and their enriched pathway in three mouse models related to depression and the regulation of the ceRNA network-mediated gene in the progression of depression. Together, our findings may be crucial for uncovering the mechanisms.

Indoleamine 2,3-dioxygenase 1 (IDO1), which is the tryptophan catabolizing enzyme, affects the nervous system through two mechanisms. The first mechanism involves tryptophan depletion through over-activation of IDO1 which increases tryptophan catabolism and thereby reduces the levels of tryptophan, as well as suppressing the synthesis of 5-HT, hence resulting in depression (Chaves Filho et al., 2018). The second mechanism is the increase in kynurenine toxicity mediated by IDO1 (Jiang et al., 2020). It has been found that although kynurenine is neuroprotective, it is neurotoxic at excessive levels.

Therefore, it is evident that the reduction of BDNF can cause depression-like symptoms in mice (Jiang et al., 2019) whereas the knockout of IDO1 has antidepressant-like effects (Gao et al., 2021). Furthermore, there is no corresponding report on the mRNA sequencing of the comparison between BDNF and IDO1, but this study sequenced the mRNA expression in BDNF^+/−^, IDO1^−/−^, chronic ultra-mild stress (CUMS), and control mice.

## Materials and Methods

### Animals and Experimental Groups

To avoid the effects of sex differences and hormones, only male mice were selected for the current study. Mice (10 per group) were randomly assigned to the control (untreated), CUMS-exposed (mimicking adult stress), BDNF^+/−^ (strain BDNF^tm1Krj/J^, C57BL6/J background, Jax Strain #006579), and IDO1^−/−^ (strain IDO1^tm1Alm/J^, Jax Strain #005867) groups. The detailed information about the mice is shown in Supplementary Table S1. They were housed in a pathogen-free, temperature-controlled environment (22 ± 1°C) and subjected to 12/12 h light/dark cycles, with ad libitum access to food and water except during model building. Animal experimental protocols in the current study were approved by the National Institutional Animal Care and Ethical Committee of Southern Medical University.

### Chronic Ultra-Mild Stress Protocol

CUMS modeling was performed, as previously described (Huang et al., 2017; Gao et al., 2018). Briefly, the protocol involved the sequential application of various mild stressors: 1) 24 h of food and water deprivation, 2) 1 h of empty bottle, 3) 17 h of 45° cage tilt, 4) overnight illumination, 5) 24 h of wet cage, 6) 5 min swimming in water at 4°C, 7) 24 h of disrupting the squirrel cage, 8) 24 h of foreign body stimulation, and 9) 4 h of restriction in movement.

### RNA Sequencing

TRIzol reagent was used to isolate RNA (Invitrogen, United States). The mRNA sequencing libraries were constructed using multiplex PCR amplification techniques. The sequencing of mRNA was carried out on the Illumina sequencing platform NextSeq 550, while the sequencing of microRNA was carried out on the Illumina sequencing platform Hiseq 4000.

### Mapping

Adaptors were removed by FastQC and Trimmomatic. The alignment of mRNA was conducted by STAR software with the reference mm10, while miRNA was aligned with data from miRBase. Downstream statistical analyses were carried out in R software.

### Differential Expression Analysis

The mRNA expression differential analysis was carried out using DESeq2. Volcano plots were plotted by the EnhancedVolcano package with a default cut-off for log2FC >|2|, and the default cut-off for *p*-value 10e-6 to highlight the top genes.

### Differential miRNA: mRNA Interaction

miRNAs were searched on multiple miRNA-mRNA databases using multiMiR. The differential miRNA–mRNA interaction was calculated by using the binomial test. FDR was also used to adjust for multiple tests.

### ceRNA Network Analysis

The potential lncRNAs targeting differentially expressed genes (DEGs) were searched on lncRNA2Target for the analysis of ceRNA. In addition, the ceRNA network of the collected miRNAs and lncRNAs was constructed and visualized by using the igraph package by querying interactions between them from multiple miRNA-lncRNA databases from multiMiR.

### Protein–Protein Interaction Network Analysis

The analysis of the protein–protein interaction (PPI) network of the mRNA DEGs was performed using the R package STRINGdb to generate an interaction table, and the interaction network was visualized by using the igraph package.

## Results

### Identification of Differentially Expressed Genes

It was found that the differences in expressed genes were highly significant between BDNF^+/−^ and IDO1^−/−^ mice, whereas there was a less evident difference in the gene expression between the CUMS and control groups.

**FIGURE 1:**
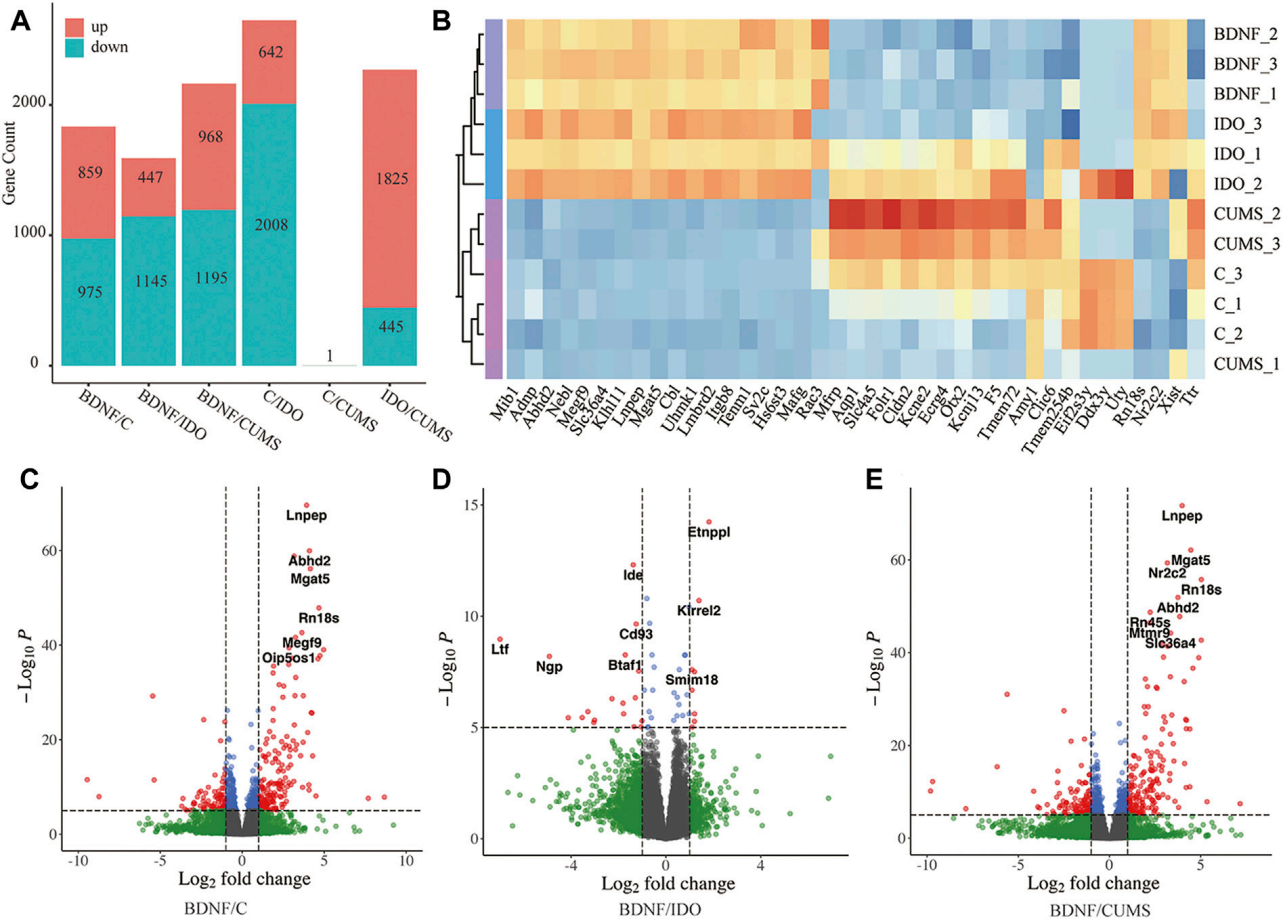
Identified DEGs in each group. **(A)** Bar plot showing statistical data of DEGs. **(B)** Sample clustering based on the expression level of top DEGs. **(C)** Volcano plot of DEGs between BDNF^+/−^ and control. **(D)** Volcano plot of DEGs between BDNF^+/−^ and IDO1^−/−^. **(E)** Volcano plot of DEGs between BDNF^+/−^ and CUMS. In the Volcano plot, blue and green scatter points represent insignificant DEGs, red scatter points represent upregulated DEGs, and blue scatter points represent downregulated DEGs. The statistical method is the default cut-off for log2FC which is >|2|, and the default cut-off for *p*-value is 10e-6 to highlight the top genes with red color.

Mouse medial prefrontal cortex (mPFC) was obtained for sequencing from BDNF^+/−^, IDO1^−/−^, CUMS-exposed, and control mice. Results of the DEG analysis revealed gene expression differences between BDNF^+/−^ and other groups, as well as modest gene expression differences in CUMS vs. control groups (Figure 1A). Consistently, the results of clustering analysis revealed close clustering between the control and CUMS samples (Figure 1B).

It was found that the analysis of gene expression identified 859 significantly upregulated and 975 significantly downregulated genes in BDNF^+/−^ vs. control samples (Figure 1A, Supplementary Table S2). Furthermore, the results of volcano plot visualization revealed that the top DEGs included Lnpep, Adhd2, Nf2c2, Mgat2, and Rn18s (Figure 1C). A heatmap with sample clustering showed the most genes that were upregulated in the expression of the top 50 different genes in BDNF^+/−^ (Supplementary Figure S1A). In addition, the results of analysis of the top five DEGs revealed that relative to BDNF^+/−^, Lnpep, Abhd2, Mgat5, Nr2c2, and Rn18s expressions were significantly higher in controls (Supplementary Figure 1B). It was evidently noted that among the DEGs, Mgat5 influences behavior and physical outcomes in response to early life stress by remodeling N-glycans and cell surface glycoproteins.

Comparison BDNF^+/−^ vs. IDO1^−/−^ identified a total of 1,145 downregulated and 447 upregulated DEGs (Figure 1A, Supplementary Table S2), including Entppl, Idem, and Kirrel2 (Figure 1D). A heatmap showed an even regulated difference among the expressions of the top 50 DEGs, indicating that IDO1^−/−^ may have a unique expression pattern under different biological mechanisms as compared with BDNF^+/−^ (Supplementary Figure S2A). It was found that the top five DEGs exhibited an evenly matched relationship between these two groups (Supplementary Figure S2B). Etnppl was evaluated as an astrocyte-specific fasting-induced gene that induces the catabolization of phosphoethanolamine (PEtN), regulating brain lipid homeostasis (White et al., 2021). The altered Etnppl expression has also been associated with mood disorders (White et al., 2021). Both genes indicated a strong change in the neural level under these two groups of models.

A comparison of BDNF^+/−^ vs. CUMS groups identified a total of 1,195 downregulated and 968 upregulated genes (Supplementary Table S2, Figure 1A). The DEGs included Lnpep, Mgat5, Rn18s, and Abdh2, which are quite similar to the results from BDNF^+/−^ vs. control (Figure 1E). Similar to the control group, the heatmap also showed the most upregulated expression in BDNF^+/−^ among the top 50 DEGs, and the top five DEGs, Abhd2, Lnpep, Mgat5, Nr2c2, and Rn18s also presented a higher expression in BDNF^+/−^ (Supplementary Figure S3). This comparison illustrated a similar result of DEGs with previous groups of BDNF^+/−^ and control, indicating that there was likely no significant difference in the gene expression between the CUMS and control groups. For the significantly different aforementioned genes , the significance threshold for statistical analysis was log2FC >|2|, and the default cut-off for *p*-value was 10e-6 to highlight the top genes with red dots.

### Pathway Enrichment Among Models

It was found that there was little difference in neural activities between BDNF^+/−^ that were involved in negative neuromodulatory pathways and IDO1^−/−^ mice, but the CUMS model did not significantly differ from controls as compared with BDNF^+/−^.

**FIGURE 2:**
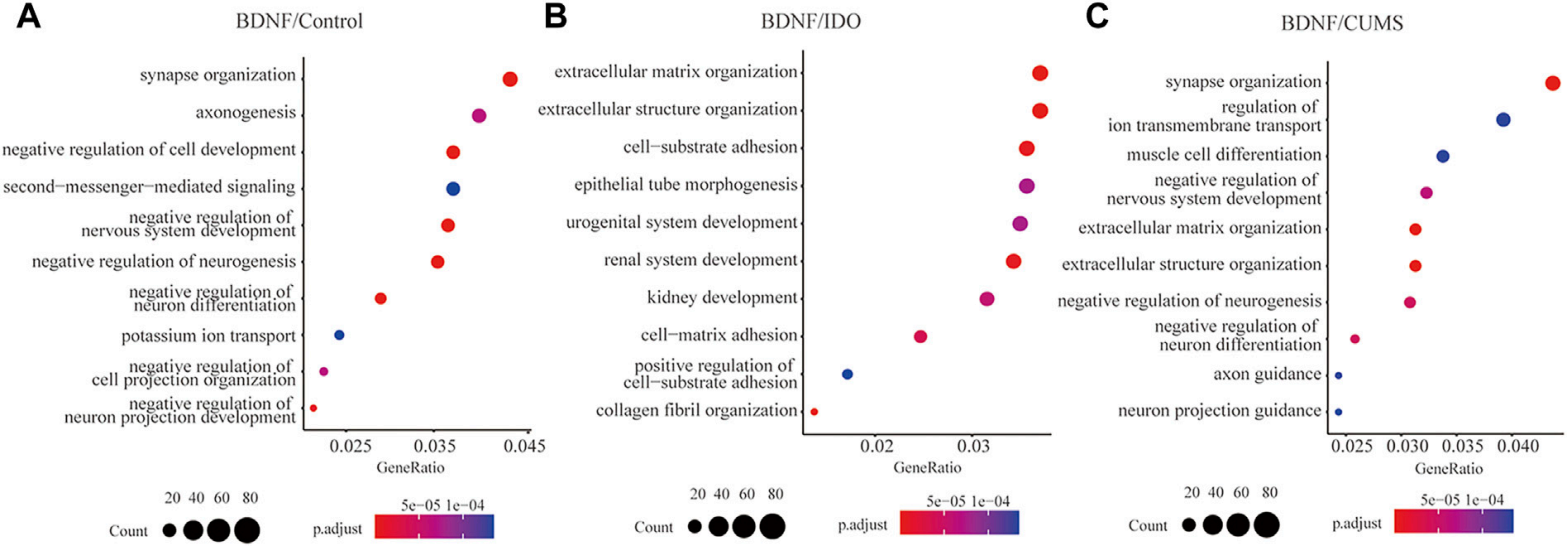
Pathway enrichment of DEGs between BDNF^+/−^ and controls. **(A)** Top 10 enriched pathways in GO terms for BDNF^+/−^ and control groups. **(B)** Top 10 enriched pathways for GO terms for BDNF^+/−^ and IDO1^−/−^ groups. **(C)** Top 10 enriched pathways for GO terms in BDNF^+/−^ and CUMS groups.

To assess pathway activation differences between the models, we subjected the DEGs to pathway enrichment analysis. Gene ontology (GO) term enrichment analysis of the BDNF^+/−^ vs. control groups identified a total of 427 pathways (Supplementary Table S3), including the negative regulation of neurogenesis, negative regulation of nervous system development, synapse organization, and negative regulation of neuron differentiation (Figure 2A), which indicated a negative neural regulation in BDNF^+/−^ mice.

The results of the heatmap and upset plot showed a common sharing gene enriched by different pathways (Supplementary Figure S7A,B). Furthermore, a comparison between the top pathways in the upset plot and their significant genes identified a high concentrated gene set that included Mib1, Foxo3, Ptbp1, Sema3c, and Sorl, enriched in a cluster of neural regulation pathways such as negative regulation of neuron differentiation, neuron projection guidance, and axonogenesis (Supplementary Figure 7C).

We identified a total of 237 significant GO terms and revealed the DEGs to be enriched for various pathways that are not related to neural regulation, including extracellular matrix organization, extracellular structure organization, collagen fibril organization, cell-substrate adhesion, and renal system development (Figure 2B, Supplementary Table S3). This indicated little difference in neural activities between BDNF^+/−^ and IDO1^−/−^ mice. The count of shared genes among top pathways was lower as compared to BDNF^+/−^ vs. control, which indicates a discrete distribution of biological functions (Supplementary Figure S8A,B).

In the top five pathways, the high concentrated gene set, including Cxcr2, Tnxb, P4ha1, Adams1, and Col4a5 among others, was not highly related to neural function (Supplementary Figure S8C). The CXCL1 chemokine deletion can cause rat depression-like behaviors, and CXCL1/CXCL2 correlates with depression-like behavior in response to chronic stress (Chai et al., 2019; Song et al., 2020).

We identified a total of 625 significant GO terms and revealed that the DEGs were significantly enriched in synapse organization, negative regulation of neuron differentiation, and negative regulation of neurogenesis (Figure 2C, Supplementary Table S3). Enriched pathways were highly associated with neural activities but slightly differed from the results of the analysis of BDNF^+/−^ vs. control which indicated that the main pathways in BDNF^+/−^ vs. CUMS and BDNF^+/−^ vs. control were the same. The current study found genes similar to those identified in BDNF^+/−^ vs. control pathway enrichment, including Mib1, Sema3c, and Foxo3, which were still enriched in relevant negative regulation of neuron activities, indicating that the CUMS model did not differ significantly from the controls as compared with BDNF^+/−^ (Supplementary Figure S9).

### Network Analysis of the Protein–Protein Interaction

PPI differences between BDNF^+/−^, a series of strong protein interactions, and IDO1^−/−^ were not focused or related to neural activities, whereas internal consistency was similar between the control and CUMS groups.

**FIGURE 3:**
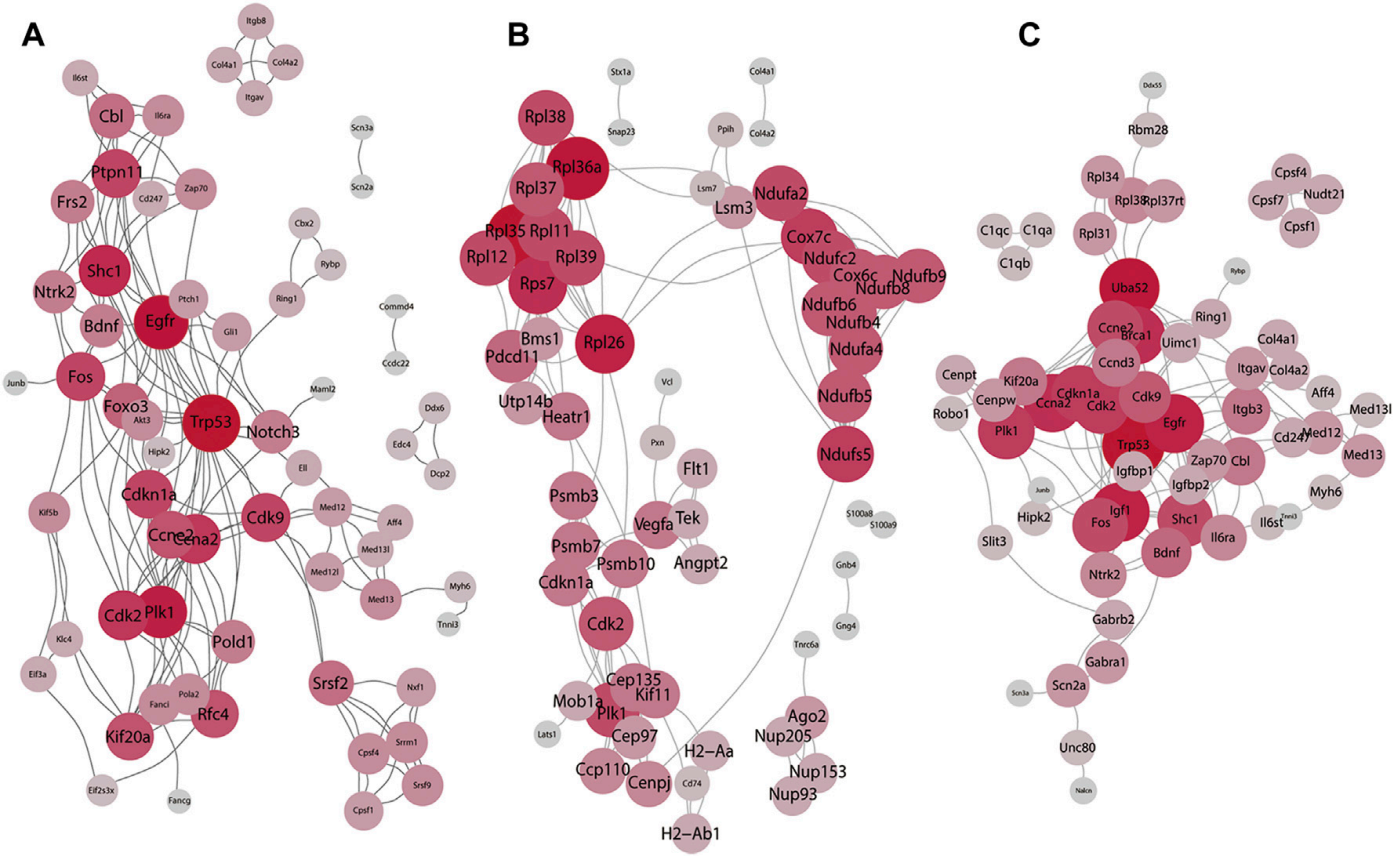
Result of the PPI network analysis. **(A)** PPI network of the top 60 DEGs in BDNF^+/−^ and control groups. **(B)** PPI network of the top 60 DEGs in BDNF^+/−^ and IDO1^−/−^ groups. **(C)** PPI network of the top 60 DEGs in BDNF^+/−^ and CUMS. The degree of red color and the size of each vertex indicate the number of connections.

To analyze the interactions with other molecules, we performed PPI based on the DEGs. Results in the BDNF^+/−^ vs. control groups and the PPI network of DEGs revealed highly confident interactions which illustrated a series of strong interactions between proteins in BDNF^+/−^ mice (Figure 3A). It was found that the whole network includes 171 links with the highest confidence among 60 nodes (score: >700). In addition, the whole network was mainly connected using several hub genes, including Trp53, Foxo3, EGFR, and CDK families. Furthermore, Trp53 responds to diverse cellular stresses to regulate target genes that induce cell cycle arrest, apoptosis, and senescence, as well as commonly interacts with CDKs which indicate cell cycle regulation changes in BDNF^+/−^ mice (Rufini et al., 2013).

In BDNF^+/−^ vs. IDO1^−/−^, it was found that the PPI network contained 178 links and 60 nodes (Figure 3B). Notably, the network had three dense subnetworks of nearly equal size. The densest was mostly composed of Rpl family genes, including Rpl36a, Rpl38, and Rpl39. Furthermore, the Rpl family is composed of L ribosomal proteins. It was found that between the other two subnetworks one was led by Cdk2, P1k1, and Psmb10, and the other was led by Ndufb6, Ndufb4, Ndufb9, and the relevant gene of the NADH dehydrogenase subunit. The three subnetworks showed a dispersion in different biological functions, indicating that PPI differences between BDNF^+/−^ vs. IDO1^−/−^ are not focused or related to neural activities.

It was found that in BDNF^+/−^ vs. CUMS, the network was composed of the top 60 DEGs with 163 interaction links (Figure 3C). The results of the PPI network revealed only one cluster of similar topology to the one in BDNF^+/−^ vs. control, as well as similar hub genes, including Trp53, EGRF, Fox, Foxo3, and CDKs, reflecting consistent similarity between control and CUMS. However, it contained other hub genes, including Uba52, Bdnf, and Zap70.

### Network Analysis of lncRNA–miRNA–mRNA ceRNA

In BDNF^+/−^ vs. control, BDNF^+/−^ vs. CUMS, and BDNF^+/−^ vs. IDO1^−/−^ mice, most differentially expressed genes were associated with the protection of vulnerable neuronal circuits. To investigate the potential interactions between DEGs and lncRNAs, we analyzed ceRNA based on DEGs among different models. For each comparison, lncRNAs and miRNAs that may interact with the DEGs were identified, and relevant interaction networks were built.

The BDNF^+/−^ vs. control lncRNA-mRNA data were obtained from lncRNA2 targets. The lncRNA-mRNA network revealed 150 interactions between 40 DEGs and 46 lncRNAs (Supplementary Figure S10A). Lnpep, Slc36a4, and Amy1 interacted with most lncRNAs whereas AK040954, Linc-RAM, H19, and Linc1388 targeted most mRNAs. The hub genes in the miRNA-mRNA network included miR-124-3p, miR-132-3p, and miR-9-5p in miRNA and Dyrk2 as well as Nr2c2 and Nbeal1 in mRNA. miR-124-3p, which had the most connections in the current study, is a well-known biomarker of neural diseases (Supplementary Figure S10B).

**FIGURE 4:**
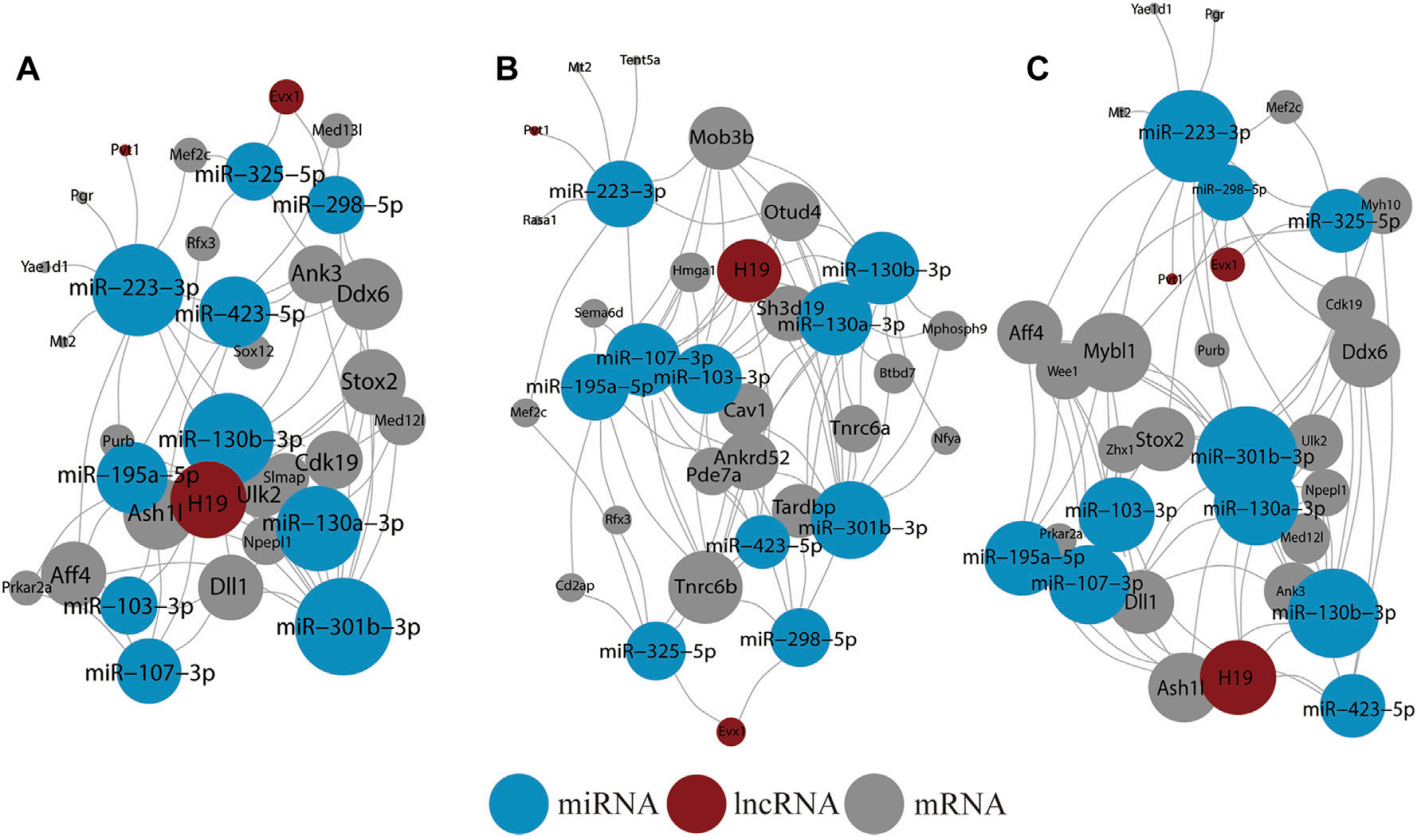
Result of ceRNA network analysis. **(A)** ceRNA network of all DEGs in BDNF^+/−^ and control groups. **(B)** ceRNA network of all DEGs in BDNF^+/−^ and IDO1^−/−^ groups. **(C)** ceRNA network of all DEGs in BDNF^+/−^ and CUMS groups. The size of the vertex indicates the number of connections.

A ceRNA network was further reconstructed (Figure 4A). In addition, it was noted that the network included lncRNAs H19, Evx1, and Pvt1, whereby H19 connected most miRNAs. The hub miRNAs included miR-130a-3p, miR-130b-3p, miR-223-3p, miR-423-5p, and miR-301b-3p whereas the hub mRNAs included Stox2, Ulk2, Npepl1, Aff4, and Ddx6.

In BDNF^+/−^ vs. IDO1^−/−^, the lncRNA-mRNA network was composed of 147 interactions between 39 DEGs and 44 lncRNAs (Supplementary Figure S11A). Myh9, Adam12, Iqgap1, and Tfrc interacted with most lncRNAs whereas linc1388, linc1382, linc1470, and linc1558 targeted most mRNAs. In the miRNA-mRNA network, miR-124-3p, miR-30e-5p, and miR-30a-5p connected with most mRNAs, whereas Ptpn13, Tfrc, Zfp36l1, and Myh9 connected with most miRNAs (Supplementary Figure S11B).

The mRNA–miRNA–lncRNA ceRNA network had three lncRNA nodes, 20 mRNA nodes, and 10 miRNA nodes (Figure 4B). The lncRNA nodes with the most connections were H19, Evx1, and Pvt1 as compared with BDNF^+/−^ vs. control. Furthermore, the miRNA nodes included miR-107-3p, miR-130b-3p, miR-130a-3p, miR-195a-5p, miR-301b-3p, and miR-103-3p. The average connection per miRNA was higher than in BDNF^+/−^ vs. control. The hub mRNAs included Tnrc6b, Mob3b, Otud4, Ankrd52m, Tardbp, Sh3d19, and Cav1, and it was found that they had little overlap with results from BDNF^+/−^ vs. control.

In BDNF^+/−^ vs. CUMS, the lncRNA-mRNA network showed 139 interactions between 37 DEGs and 46 lncRNAs (Supplementary Figure S12A). Lnpep, Slc36a4, and Amy1 still interacted with most lncRNAs, whereas AK040954, Linc-RAM, H19, and Linc1388 targeted most of the mRNAs. In the miRNA-mRNA network, hub miRNAs included miR-124-3p, miR-106-5p, miR-132-3p, and miR-9-5p, whereas hub mRNAs included Dyrk2, Nr2c2, Nbeal1, and Ptbp1 (Supplementary Figure S12B). In the mRNA–miRNA–lncRNA network, lncRNA nodes still included H19, Evx1, and Pvt1, with H19 still having the most connections (Figure 4C). Furthermore, the hub miRNAs included miR-301b-3p, miR-223-3p, miR-130a-3p, miR-130b-3p, and miR-223-3p whereas the hub mRNA gene included Mybl1, Ddx6, Aff4, Stox2, and Ddx6, which was similar to BDNF^+/−^ vs. control.

**FIGURE 5:**
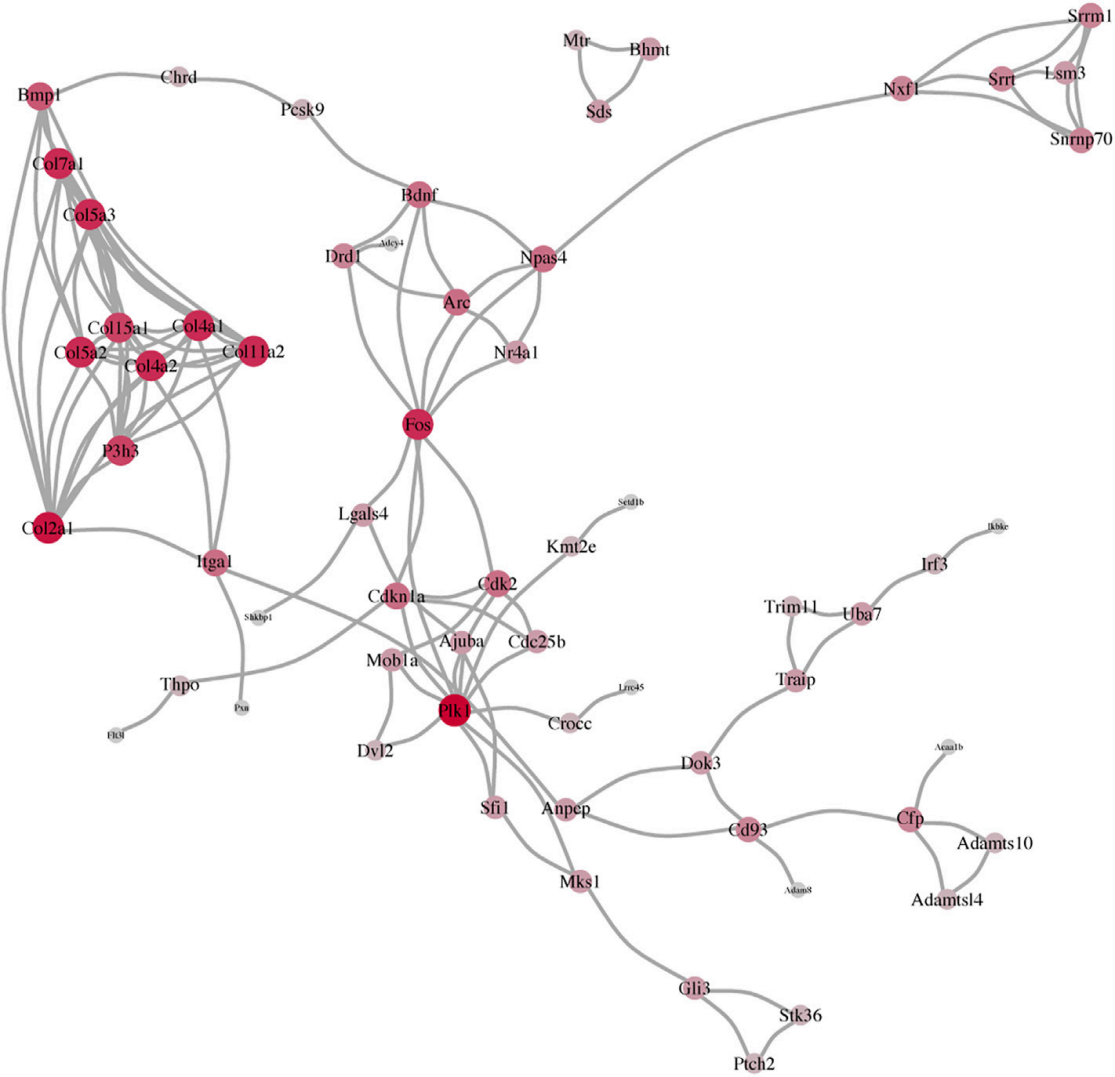
Consistency of the three PPI networks, including BDNF^+/−^ vs. control, BDNF^+/−^ vs. IDO1^−/−^, and BDNF^+/−^ vs. CUMS.

Following the consistency of the aforementioned three PPI networks, we determined the mRNA network of the BDNF^+/−^ vs. control, BDNF^+/−^ vs. IDO1^−/−^, and BDNF^+/−^ vs. CUMS mice. It showed that BDNF was a common difference between them, which was also in line with the differential expression of the prefrontal lobe after the knockdown of the Bdnf. Among them, we found that not only was the upstream Bmp1 of Bdnf different but also the downstream Fos of Bdnf and Fos was also an important indicator of activating neuronal activity (Figure 5).

## Discussion

The current study identified several differentially expressed genes in normal vs. depression-like mouse tissues from diverse genomic locations. These genes were collected in an mPFC manner. Pathway enrichment and ceRNA network analyses evidently revealed that most differentially expressed genes were associated with the protection of vulnerable neuronal circuits, and enriched pathways were associated with nervous system development and synapse organization.

Consistent with several previous studies, it was found that there were no significant gene expression differences in control vs. CUMS mice (Ma et al., 2016; Ma et al., 2019). It was evident that the possible differences are not reflected at the transcriptomic level but in protein modification or neurotransmitter content. However, it was found that BDNF-knockdown mice exhibited depression-like features based on reduced levels of neurotransmitter content (Kojima et al., 2020). Furthermore, it was found that the BDNF^+/−^ mice exhibited significant gene expression differences as compared with control or IDO1^−/−^ mice.

It was also evident that various genes, including Ptbp1, were predominantly expressed in BDNF^+/−^ as compared with other groups which suggested that they were purposefully produced. This study focused on mouse mPFC sequencing of gene modification, especially in BDNF^+/−^ and IDO1^−/−^ mice. Other previous studies have reported more differential mRNA expressions in the hippocampus, and there are possibilities of molecular lateralization in other subcortical areas (Hu et al., 2020; Chae et al., 2021). Furthermore, various abundant genes are specifically expressed in the gene-editing group and differentially expressed in the depression-like group as compared with the normal or depression-like antagonism groups, hence indicating that they serve specific functions in specific pathways (Le et al., 2018; Xu et al., 2019).

The current study had some limitations. The first limitation was the lack of sequencing comparison between other brain regions such as the hippocampus of the limbic system or the parahippocampal gyrus and cingulate gyrus. The lack of comparison of human samples was also a shortcoming of this study. Adding human-derived depression samples would have enriched the understanding of the degree of gene expression changes in depression-like lesions. Overall, the current study only performed RNA sequencing studies on a CUMS-based depression-like mouse model, BDNF knockdown mice (simulating depression-like), and IDO knock-out mice (antagonizing depression-like). The sample area was the prefrontal cortex, and because no human samples were analyzed for comparison in the current study, there was no experimental verification of whether the differential gene expression, including Ptbp1, is associated with depression pathogenesis.

Nevertheless, the results of the current study suggest that in a mouse model of depression (BDNF^+/−^), CXCL1 deletion (Chai et al., 2019) and Slc17a7 reduction (Lindstrom et al., 2020) are related to the loss of excitatory neurons in the prefrontal lobe, whereas Ptbp1 downregulation (Qian et al., 2020) correlates with neuronal regeneration. However, there is a need for experimental validation of these findings in future research.

## Conclusion

Depression mouse models and controls were studied for possible DEGs and enriched pathways. The findings show a function for ceRNA network-mediated genes in the development of depression. There is a difference in the expression between BDNF^+/−^ and CUMS model depressed mice, showing that the BDNF knockout model can only assist in imitating neurotransmitter models. A neurotransmitter disruption was not seen in the IDO1^−/−^ mouse model, in contrast to the CUMS and BDNF^+/−^ models. Our findings may help unravel the neurotransmitter hypothesis of depression in animals.

## Data Availability

The data presented in the study are deposited in the NCBI repository, accession number PRJNA825298.
