# Supplementary material for: Identifying Antidepressant Effects of Brain-Derived Neurotrophic Factor and IDO1 in the Mouse Model Based on RNA-Seq Data
Source: Front Genet. 2022 May 30;13:890961. doi: 10.3389/fgene.2022.890961 (PMC9195421; doi:10.3389/fgene.2022.890961)
Supplement: Supplementary file 3 [file DataSheet1.PDF]

Supplementary Figure 1

A

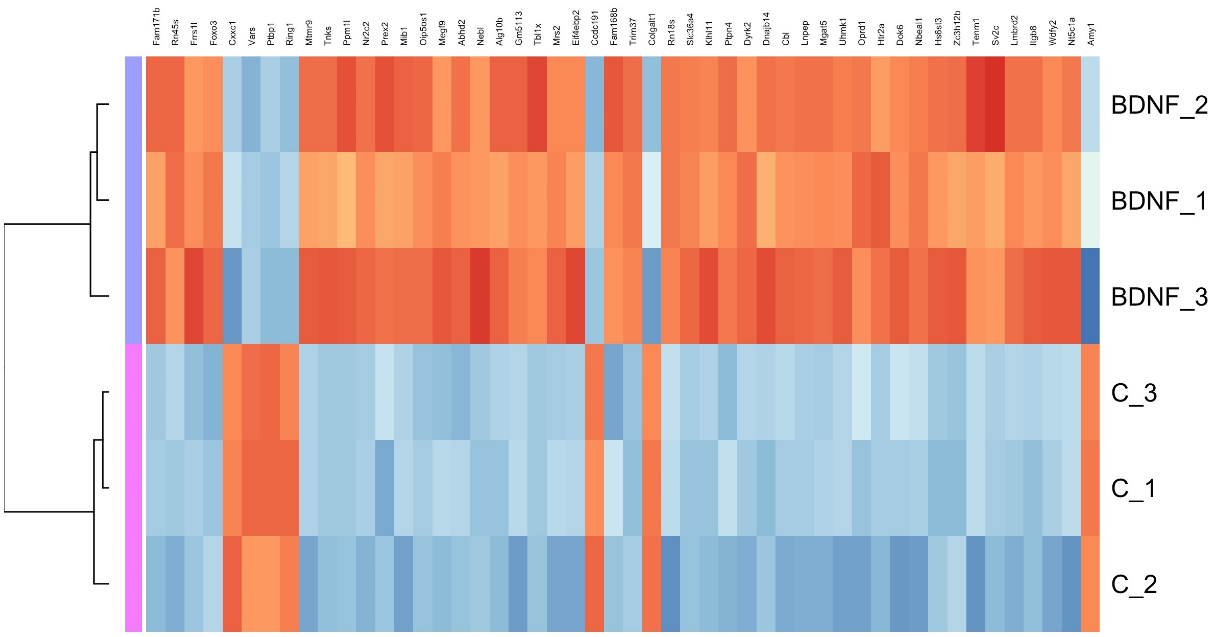

B

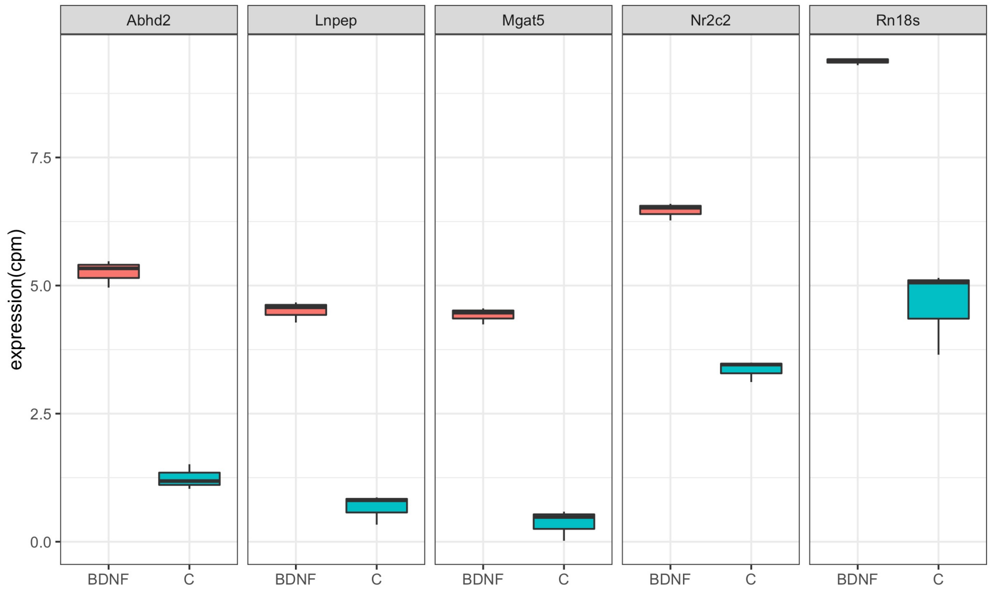

DEGs result of BDNF vs Control. (A) Heatmap of top 50 DEGs . (B) Barplot of Top 5 differentially expressed mRNA.

Supplementary Figure 2

A

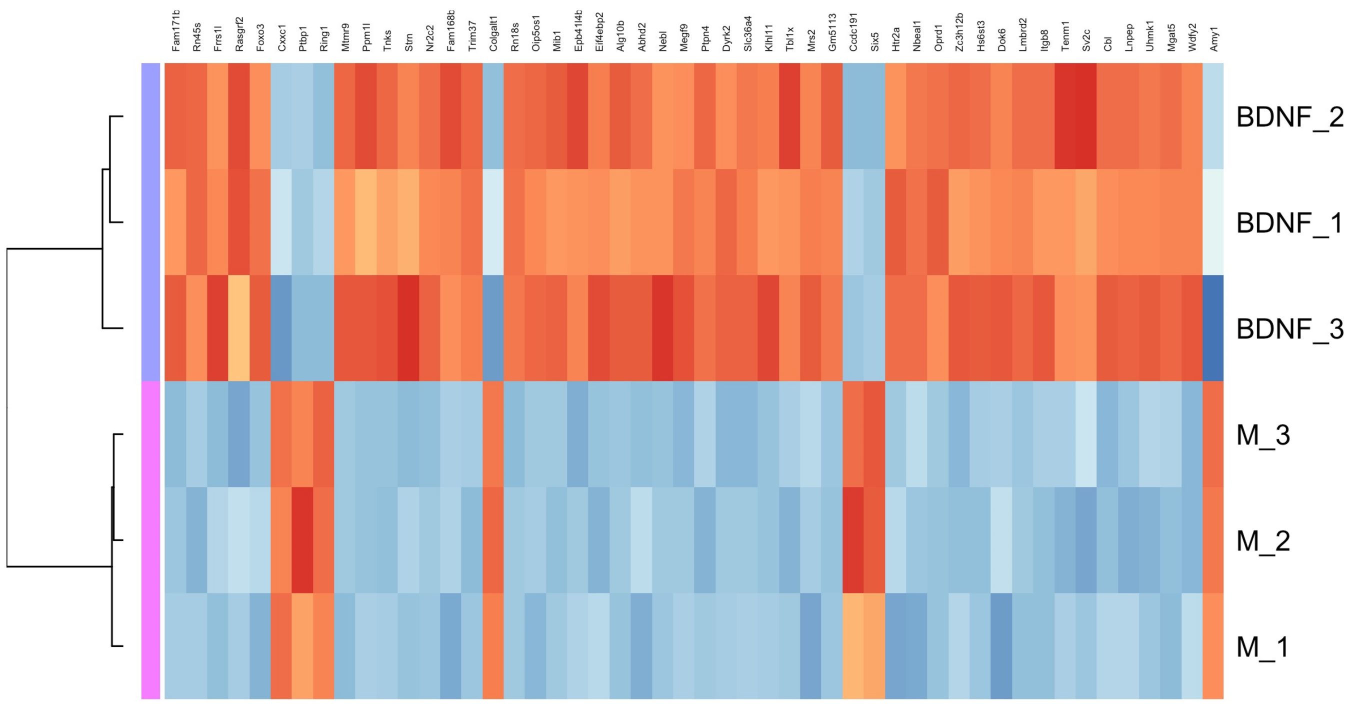

B

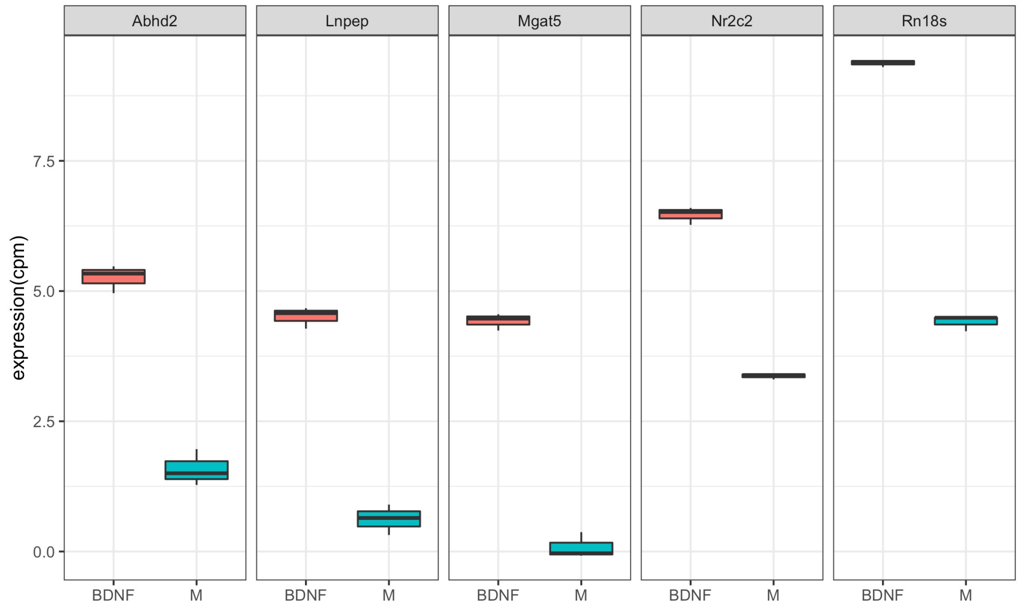

DEGs result of BDNF vs IDO. (A) Heatmap of top 50 DEGs . (B) Barplot of Top 5 differentially expressed mRNA.

Supplementary Figure 3

A

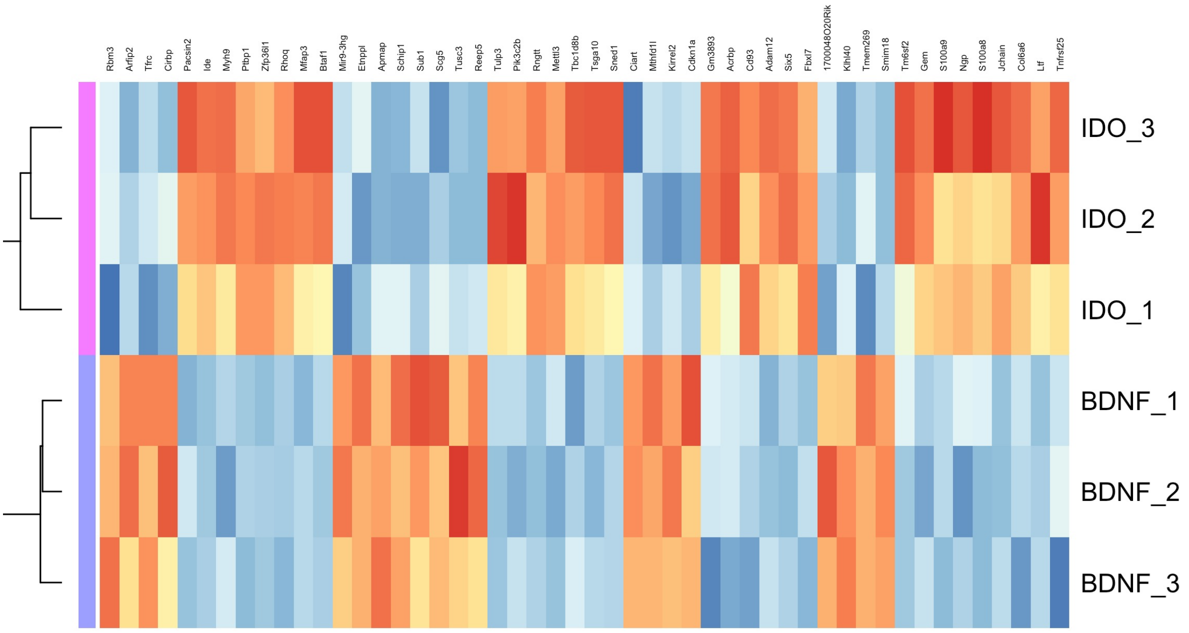

B

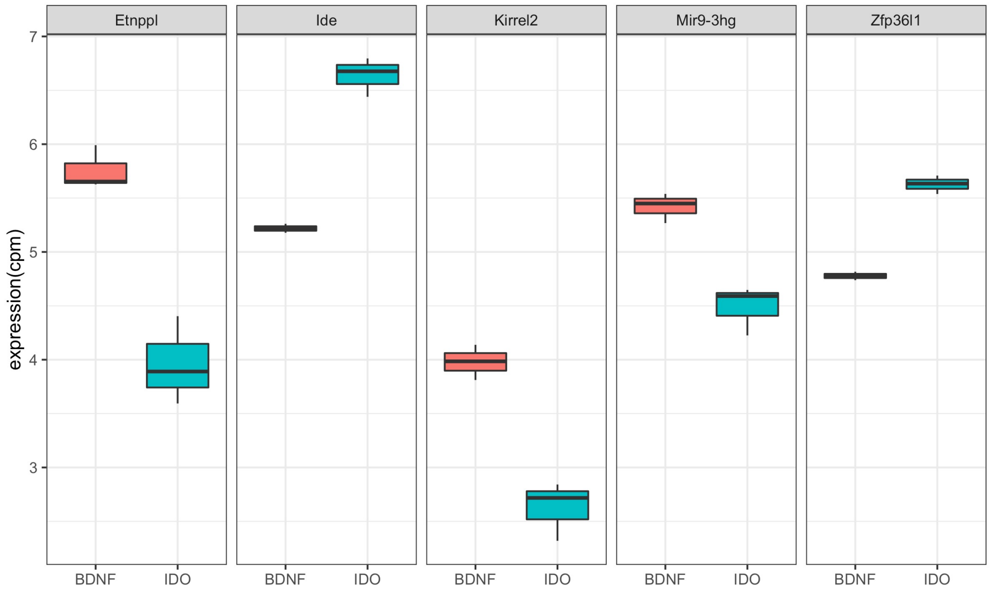

DEGs result of BDNF vs CUMS. (A) Heatmap of top 50 DEGs . (B) Barplot of Top 5 differentially expressed mRNA.

Supplementary Figure 4

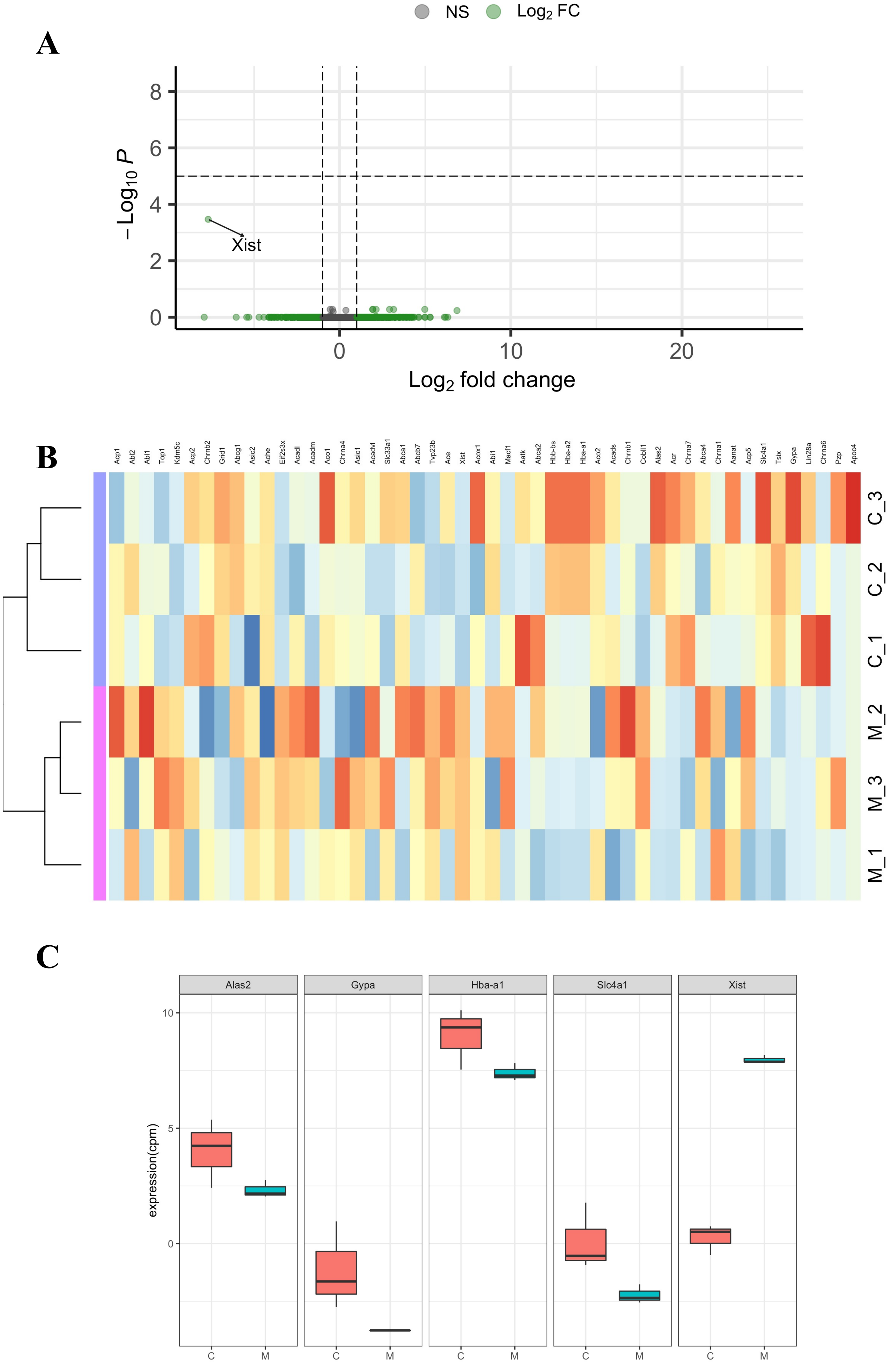

Result of DEGs in CUMS vs Control. **(A) (C)** Barplot of Top 5 differentially expressed mRNA in BDNF vs Control.

Supplementary Figure 5

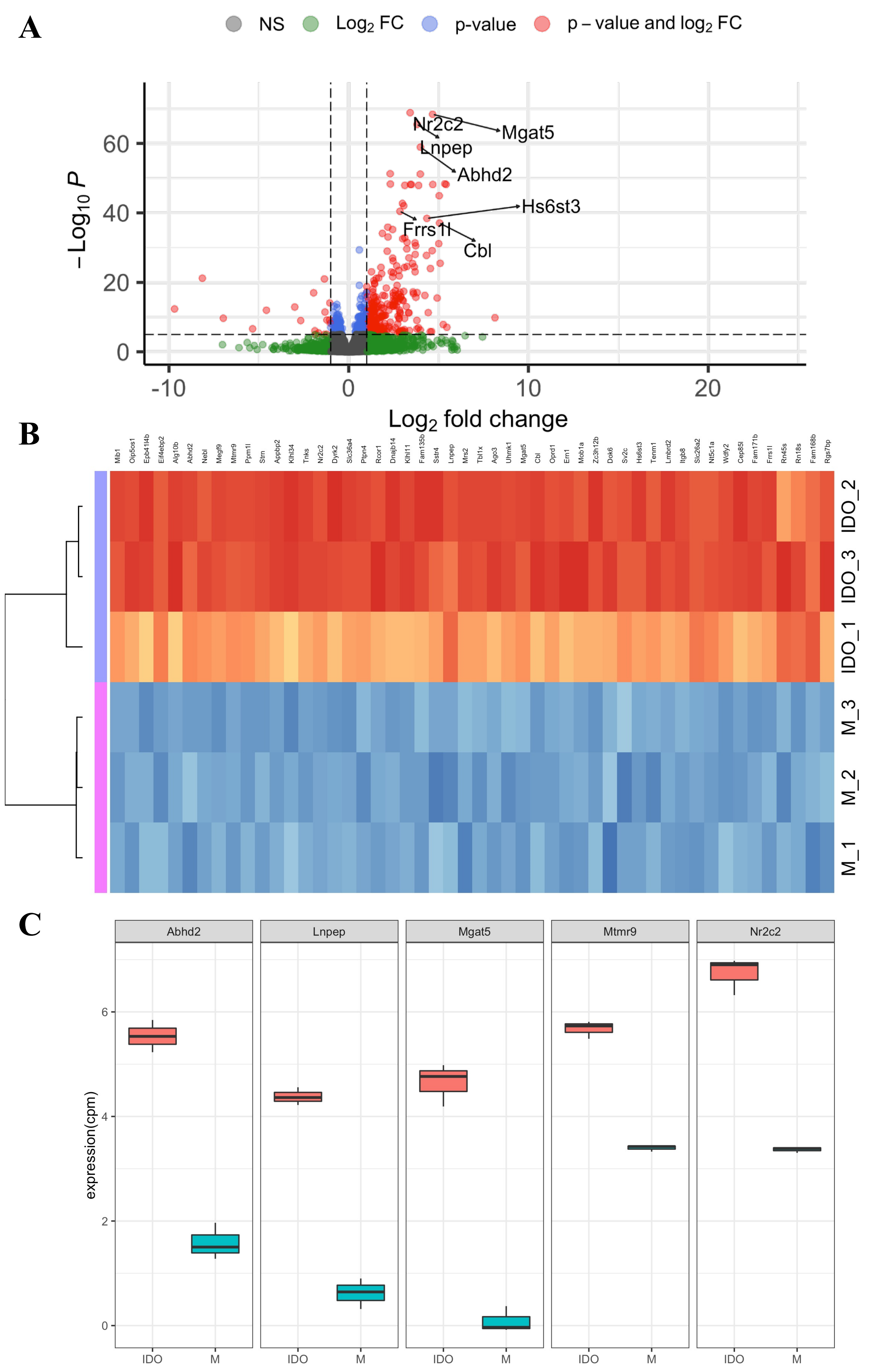

Result of DEGs in CUMS vs IDO. **(A)** Volcano plot of DEGs. **(B)** Heatmap with samples clustered. **(C)** Barplot of Top 5 differentially expressed mRNA in BDNF vs Control.

Supplementary Figure 6

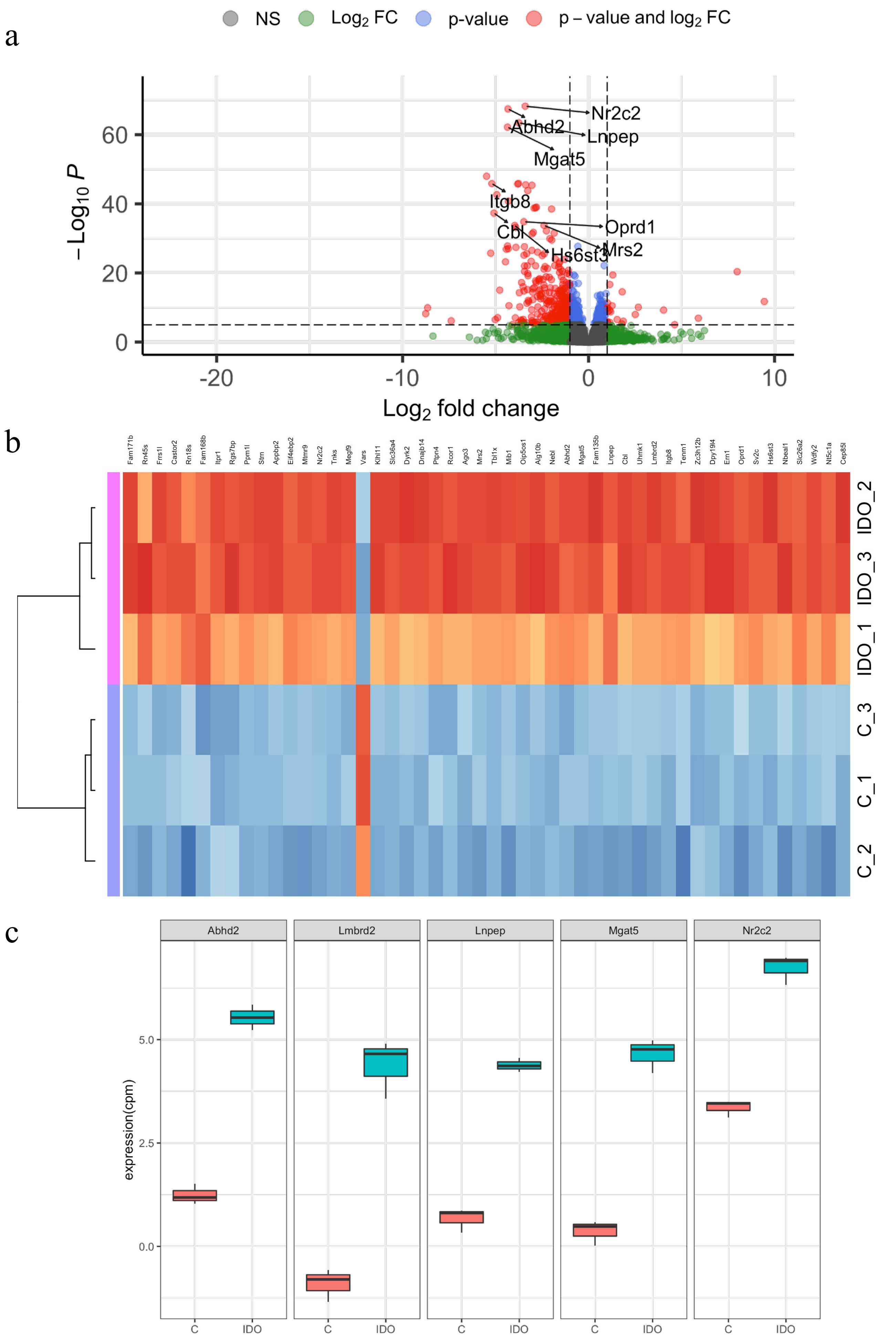

Result of DEGs in IDO vs Control**.** **(A)** Volcano plot of DEGs. **(B)** Heatmap with samples clustered. **(C)** Barplot of Top 5 differentially expressed mRNA in BDNF vs Control.

Supplementary Figure 7

BDNF vs Control

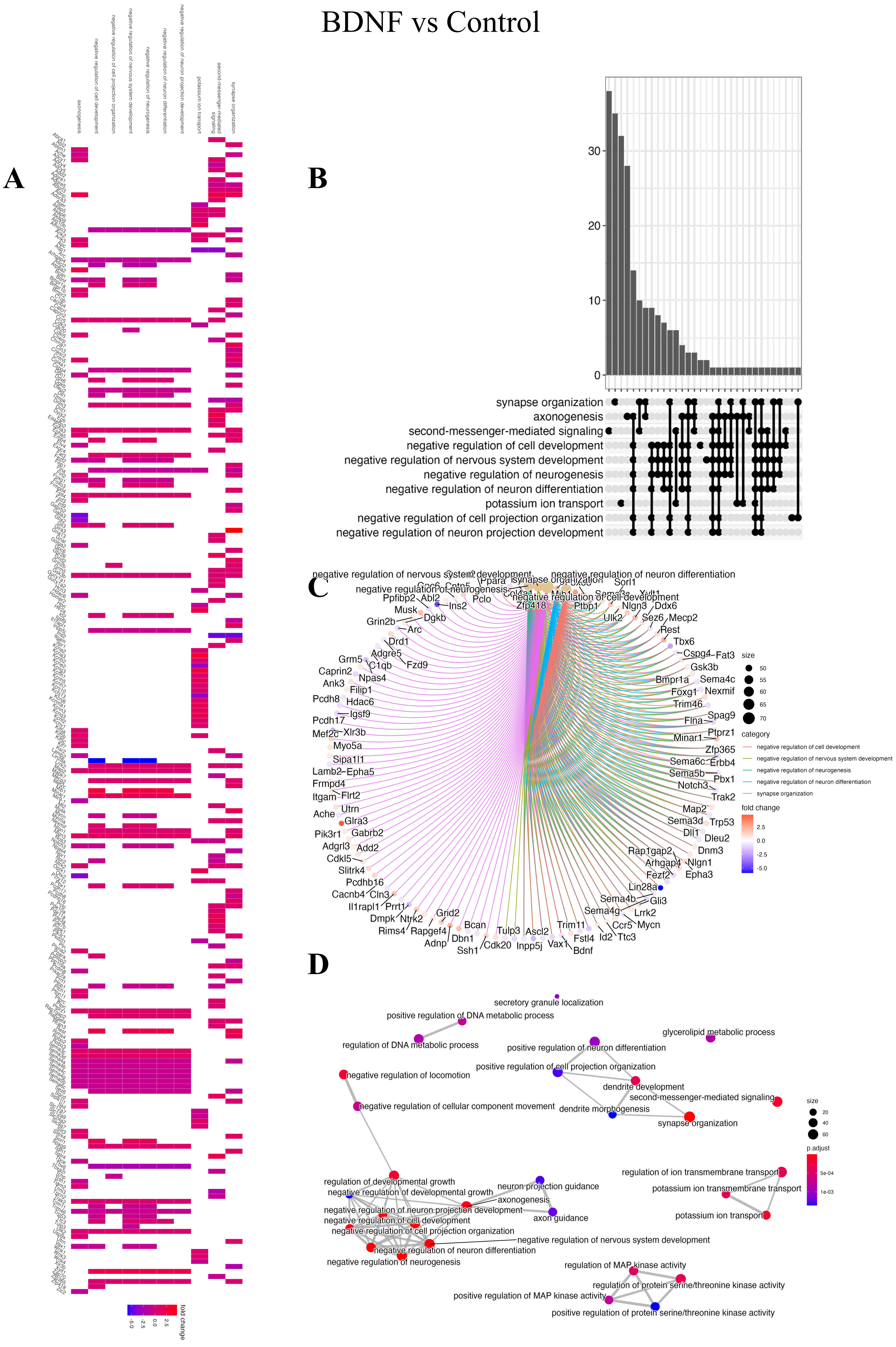

Result of pathway enrichment between BDNF and Control. **(A)** Circle plot shows Top 5 GO pathways with relevant genes and overlap. **(B)** Heatplot of gene expression in top GO pathways. **(C)** Upsetplot of top GO pathways with genes sharing statistics. **(D)** network plot of top 30 pathways.

Supplementary Figure 8
BDNF vs IDO

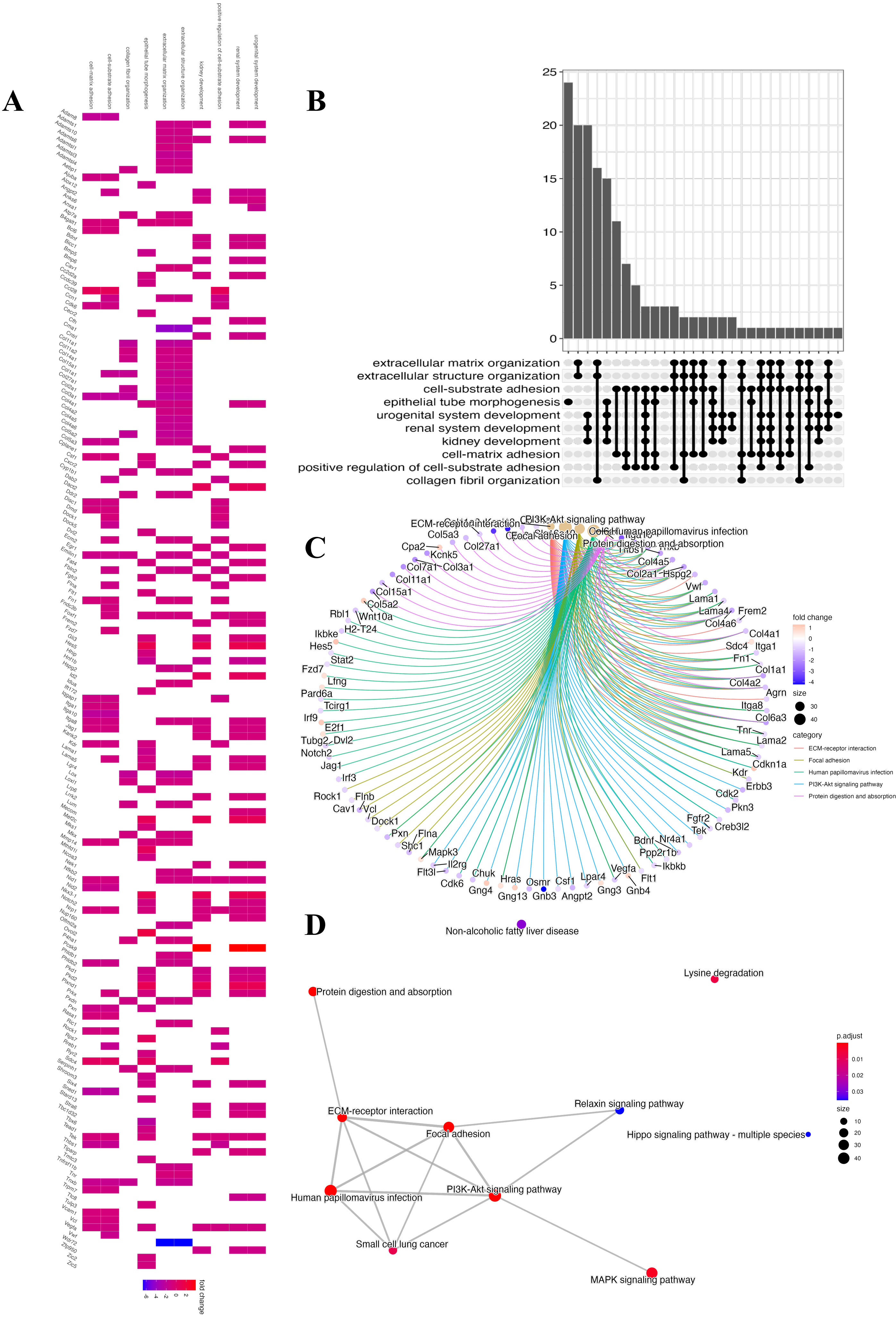

Result of pathway enrichment between BDNF and IDO. **(A)** Circle plot shows Top 5 GO pathways with relevant genes and overlap. **(B)** Heatplot of gene expression in top GO pathways. **(C)** Upsetplot of top GO pathways with genes sharing statistics. **(D)** network plot of top 30 pathways.

Supplementary Figure 9

BDNF vs IDO

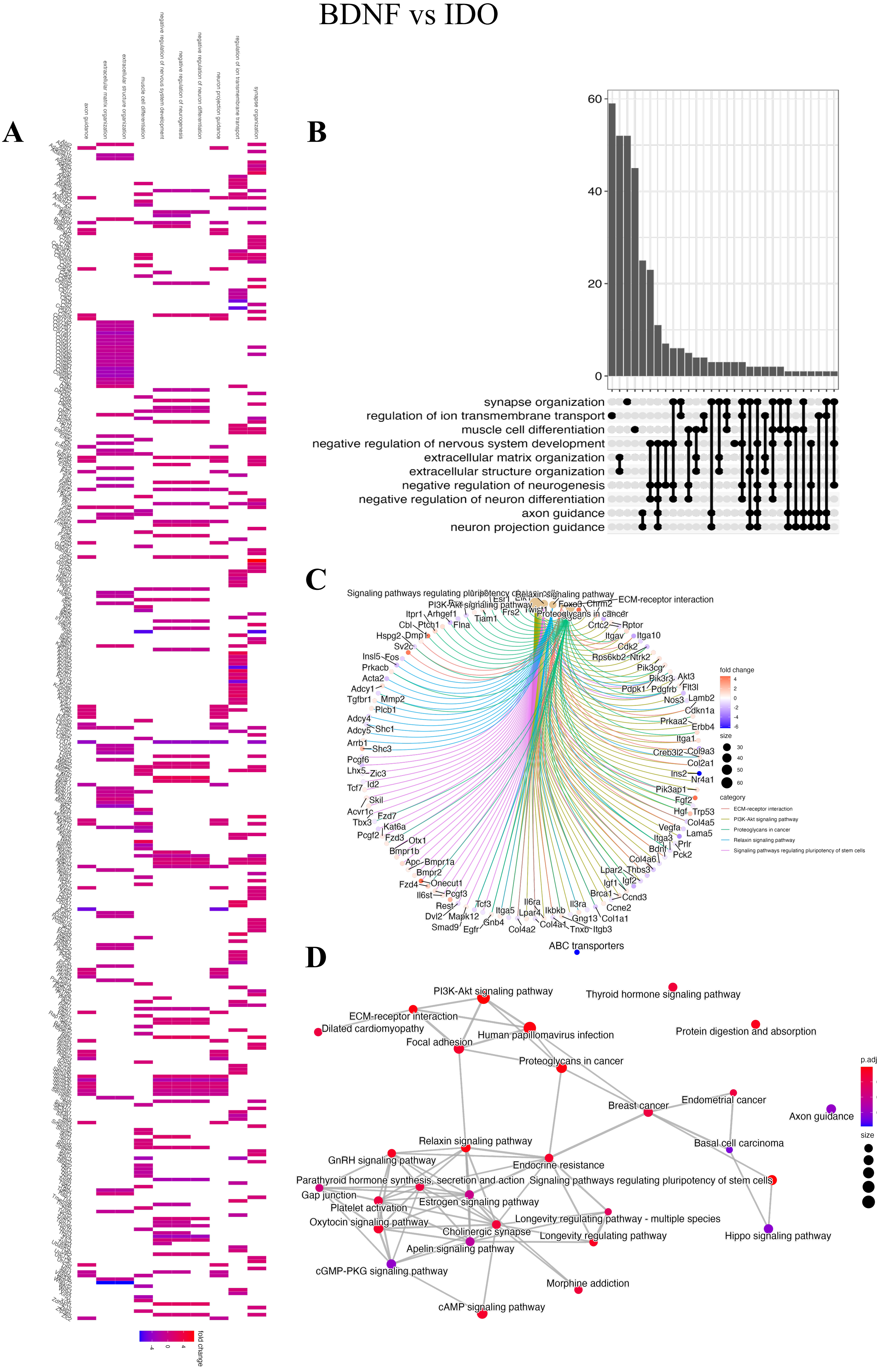

Result of pathway enrichment between BDNF and CUMS. **(A)** Circle plot shows Top 5 GO pathways with relevant genes and overlap. **(B)** Heatplot of gene expression in top GO pathways. **(C)** Upsetplot of top GO pathways with genes sharing statistics. **(D)** network plot of top 30 pathways.

Supplementary Figure 10

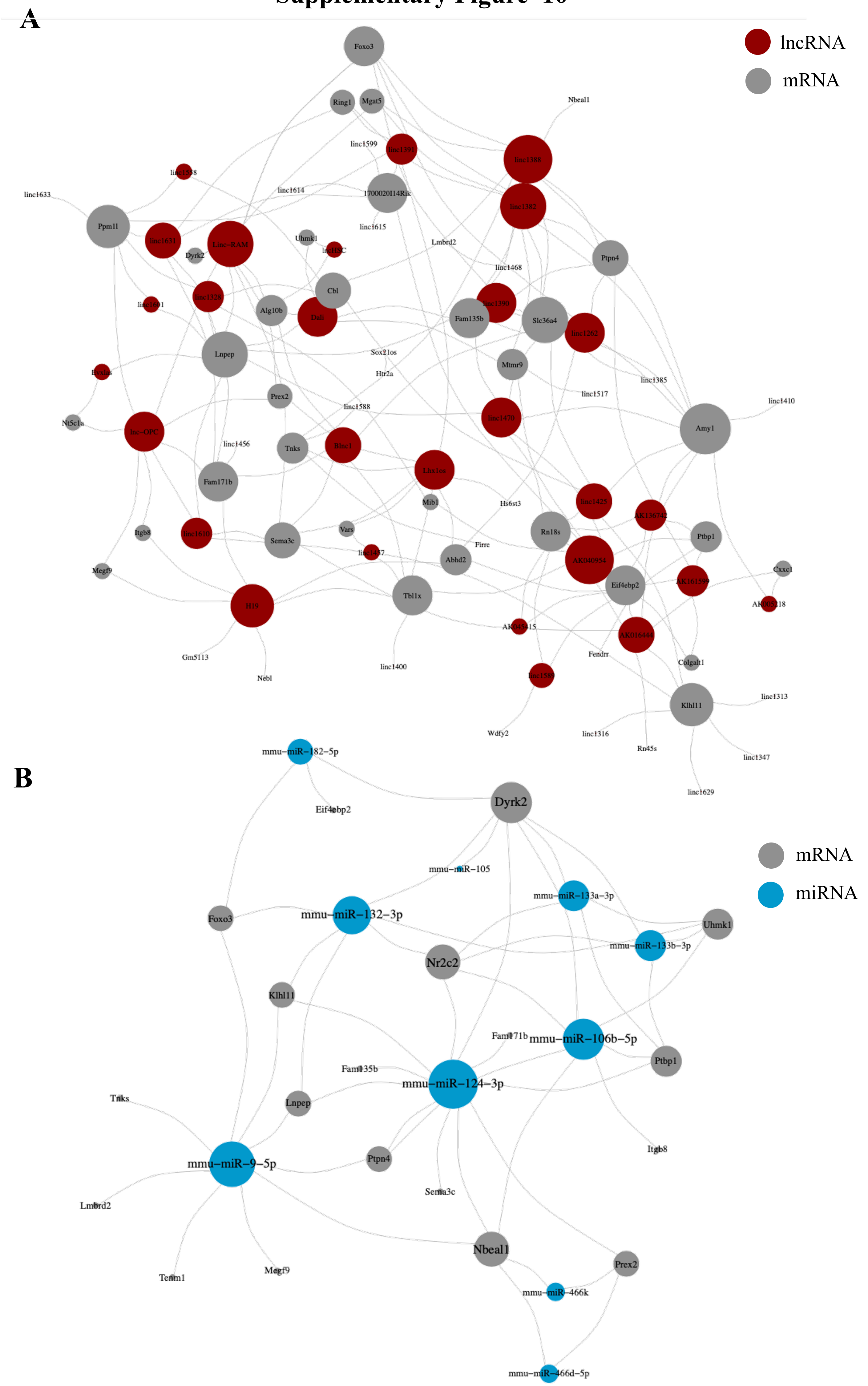

Result of network analysis between BDNF and Control. **(A)** Networks of. **(B)** networks of miRNA-mRNA

Supplementary Figure 11

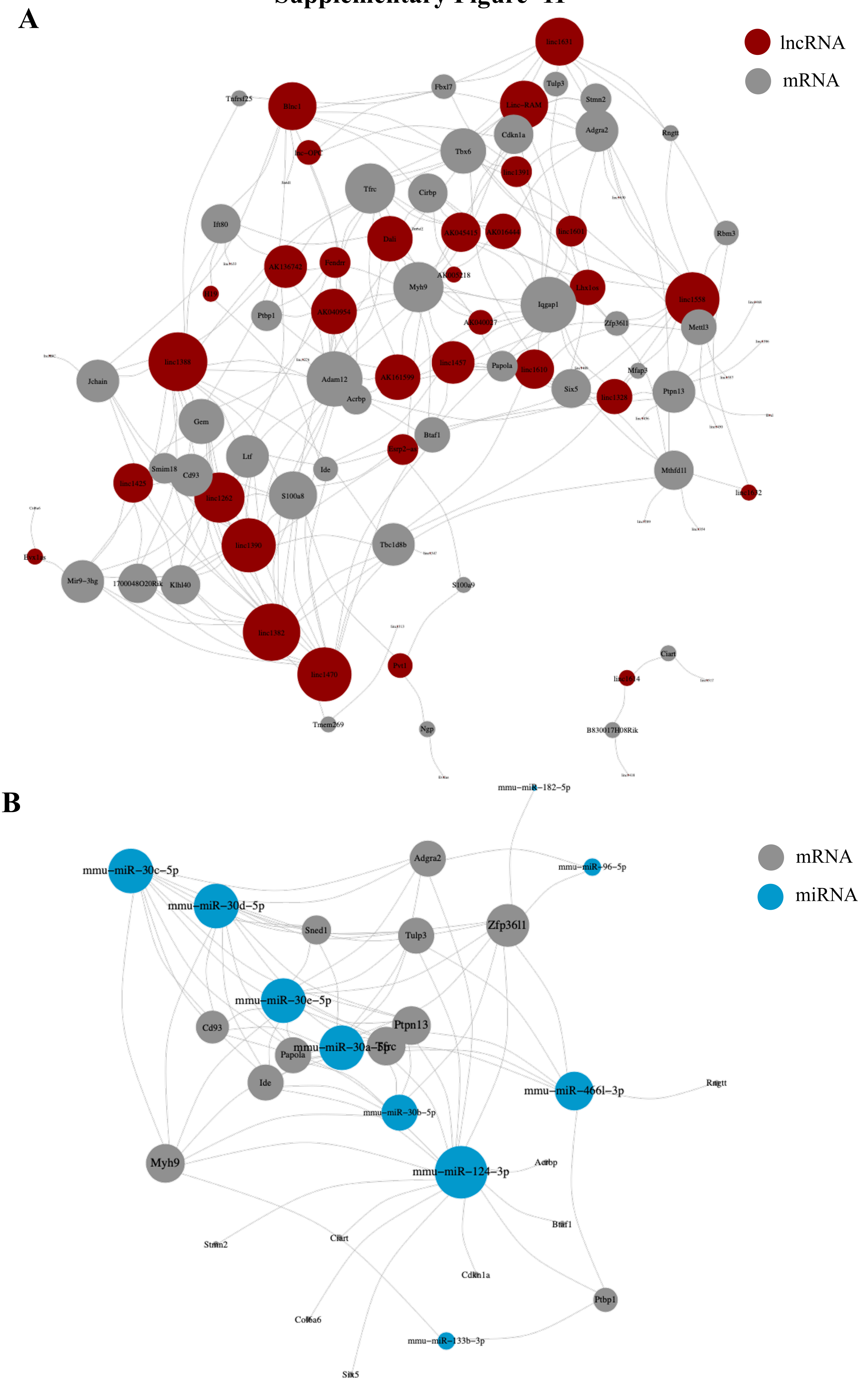

Result of network analysis between BDNF and IDO. **(A)** Networks of. **(B)** networks of miRNA-mRNA

Supplementary Figure 12

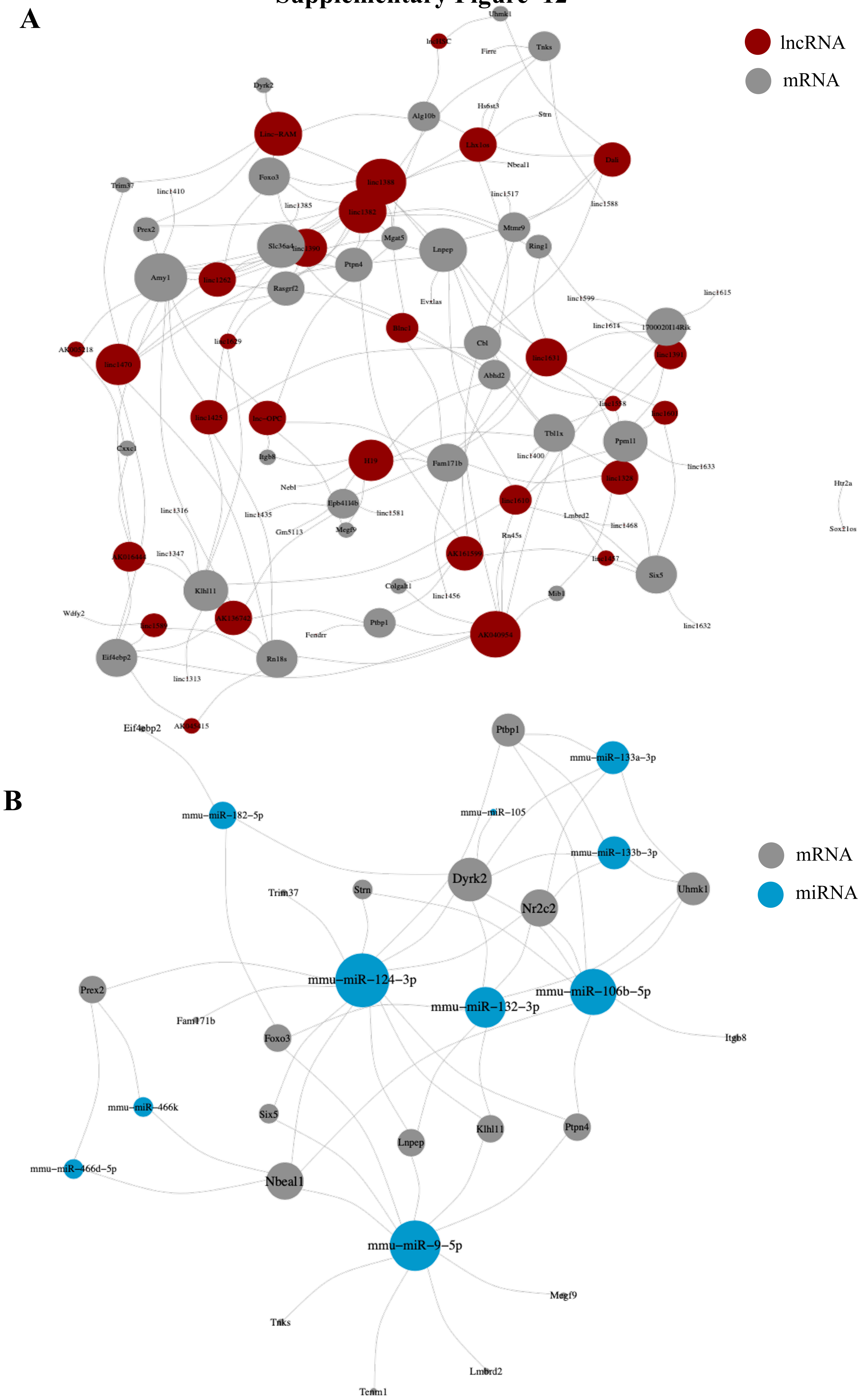

Result of network analysis between BDNF and CUMS. **(A)** Networks of. **(B)** networks of miRNA-mRNA
